# An Intelligent Fault Diagnosis Method for Reciprocating Compressors Based on LMD and SDAE

**DOI:** 10.3390/s19051041

**Published:** 2019-02-28

**Authors:** Yang Liu, Lixiang Duan, Zhuang Yuan, Ning Wang, Jianping Zhao

**Affiliations:** College of Safety and Ocean Engineering, China University of Petroleum, Beijing 102249, China; llxxyy1002@163.com (Y.L.); yuanz1007@163.com (Z.Y.); 18642167839@163.com (N.W.); 13261590998@163.com (J.Z.)

**Keywords:** reciprocating compressor, deep learning, stack denoising autoencoder, local mean decomposition, fault diagnosis

## Abstract

The effective fault diagnosis in the prognostic and health management of reciprocating compressors has been a research hotspot for a long time. The vibration signal of reciprocating compressors is nonlinear and non-stationary. However, the traditional methods applied to processing such signals have three issues, including separating the useful frequency bands from overlapped signals, extracting fault features with strong subjectivity, and processing the massive data with limited learning abilities. To address the above issues, this paper, which is based on the idea of deep learning, proposed an intelligent fault diagnosis method combining Local Mean Decomposition (LMD) and the Stack Denoising Autoencoder (SDAE). The vibration signal is firstly decomposed by LMD and reconstructed based on the cross-correlation criterion. The virtual noise channel is constructed to reduce the noise of the vibration signal. Then, the de-noised signal is input into the trained SDAE model to learn the fault features adaptively. Finally, the conditions of the reciprocating compressor valve are classified by the proposed method. The results show that classification accuracy is 92.72% under the condition of a low signal-noise ratio, which is 5 percentage points higher than that of the traditional methods. This shows the effectiveness and robustness of the proposed method.

## 1. Introduction

Reciprocating compressor units are widely used in the petroleum and chemical industries for gas pressurization and transportation. As a key piece of equipment, the running state directly affects the normal operation of the whole production system. Due to the complex structure and so many vulnerable parts of reciprocating compressor, the failure rate is always high, and the valve is the component with the highest failure rate in reciprocating compressor. Once a fault occurs, it will cause dangerous gas to leak, thus resulting in economic losses, disastrous accidents, and even possibly pose a threat to the personal safety of employees [1]. The technology of Prognostics and Health Management (PHM) can not only avoid the fatal failure of the machine but also improve the safety and the reliability of the system. PHM generally includes the ability of fault diagnosis, fault prognostics and health management; fault diagnosis is one of the most important applications in PHM. Therefore, research on the fault diagnosis of reciprocating compressors has important theoretical and practical values.

Many fault diagnosis methods have been widely studied in recent years, and some achievements have been made [2,3,4,5]. Chen et al. [2] proposed a novel method to design adaptive undecimated lifting scheme packet (AULSP), and this method was applied to identify successfully weak-signal fault features of a certain reciprocating compressor. Tang et al. [3] established a time-frequency distribution algorithm based on the concept of local frequency (LF), and applied it to the fault feature extraction of a reciprocating compressor gas valve vibration signal. Kocyigit et al. [4] removed the gap between the information required to apply a general theory of diagnosis and the limited information on the p-h diagram, which can interpret more failures of the vapor compression refrigeration cycle. Cui et al. [5] proposed a method of compressor valve fault diagnosis using information entropy and SVM and this method shows good performance in compressor valve fault diagnosis. However, there are still three obvious problems. (1) Signal processing methods are widely used; wavelet analysis [6,7,8] and Empirical Mode Decomposition (EMD) [9,10], have achieved good results. However, due to the non-stationary and nonlinear characteristics of reciprocating compressors’ vibration signals, the phenomenon that the system signals and noise signals overlap each other in the frequency band are often unavoidable, which makes the traditional method to have great limitations when dealing with the vibration signals, and the effects are often not ideal. (2) The traditional feature extraction methods [11,12,13,14] extract features artificially from the time domain or frequency domain signals with complex signal processing and subjective selection of multiple variables. Therefore, these methods usually have subjective impacts on the diagnosis results for prior knowledge and heavy dependence on signal processing technologies. (3) The traditional machine learning-based classification algorithms face the problems of dimensional disaster and overfitting [15,16] since these methods usually learn the low-dimensional shallow features and lack the necessary generalization abilities [17].

In recent years, signal adaptive decomposition methods have become a new research hotspot in the field of fault feature extraction. They are especially suitable for extracting features from signals with non-stationary and nonlinear characteristics and have achieved considerable research results in engineering applications. Among them, LMD is a signal adaptive decomposition method [18]. This method decomposes a complex non-stationary signal into the sum of several Product Function (PF) components. The complete time and frequency distribution of the original signals are obtained by superimposing the instantaneous frequency and instantaneous amplitude of all PF components. Further analysis of the original signal can better understand the state of the device, and it has been successfully applied to the time-frequency analysis of electroencephalography signals. As an ideal non-stationary signal processing method, LMD now plays a powerful role in the fault diagnosis of rotating machinery [19,20,21], with the advantages of reducing the endpoint effect, reducing the inaccuracy of enveloping, and preserving information. Some researchers have introduced the concept of LMD to the fault diagnosis of reciprocating compressors [22,23,24]. Although this method reduces the endpoint effect and inaccuracy of the envelope, the follow-up studies are still based on conventional methods for vibration signal analysis and feature extraction. 

Auto-encoder neural networks based on deep learning are a hot topic in data dimensionality reduction and feature extraction. As an efficient pattern recognition network system, deep learning has potential advantages in current intelligent fault diagnosis [25]. Several Deep Learning methods, such as the Convolutional Neural Network (CNN) [26,27,28,29], the Deep Belief Network (DBN) [30,31,32,33] and the Auto-encoder (AE) and its derivative algorithm [34,35,36], have been applied in fault detection and diagnosis. Deep learning is characterized by stacking multiple levels of a deep network structure in the network to fully explore the collected signal information. Massive data can be used to learn effective features through multiple linear and nonlinear transformations for machine health condition classification [37]. Therefore, deep learning is more effective than the traditional classification algorithms, such as the Support Vector Machine (SVM) and the Clustering algorithm, which can only identify shallow structures.

In view of the three problems of the existing methods mentioned above, this paper, based on the idea of LMD and deep learning, proposes an intelligent fault diagnosis method that combines Local Mean Decomposition (LMD) and the Stack Denoising Autoencoder (SDAE) for reciprocating compressor diagnosis. The proposed method consists of three consecutive stages. First, the vibration signals are decomposed by LMD and the cross-correlation between the PF components and the reference signal is calculated based on the cross-correlation criterion to reconstruct the signal. Then, the model of the SDAE is trained to learn the data hierarchically and automatically learn the fault information of the input signal. Finally, the automatic feature extraction and fault classification of mechanical equipment are realized by the proposed method. To verify the effectiveness and the robustness, the proposed method is compared with the traditional methods for the feature extraction and accuracy of fault classification. The main contributions of this article are as follows. (1) The vibration signal is decomposed adaptively by LMD, and the cross-correlation is calculated based on the cross-correlation criterion to reconstruct the signal to solve the problem with the overlapping of the system signal and the noise. (2) The denoised signal is input into the trained SDAE model, and the features are extracted automatically, which solve the subjectivity problem of manual feature extraction. (3) Aiming at the problem of the dimension disaster and over-fitting in traditional machine learning-based classification algorithms, an intelligent fault diagnosis method combining LMD and SDAE is proposed. 

The remainder of this paper is organized as follows. Section 2 briefly introduces the theoretical background for LMD and SDAE. Section 3 describes the intelligent fault diagnosis method of reciprocating compressor based on LMD and SDAE. Section 4 presents the experimental results and analysis demonstration using the proposed method. In addition, the effectiveness and robustness of the proposed method are verified by compared with the traditional methods. Finally, Section 5 gives the conclusions.

## 2. Theoretical Background

### 2.1. Local Mean Decomposition

The LMD method essentially separates the pure frequency modulation signal and the envelope signal from the original signal. By multiplying the pure frequency modulation signal and the envelope signal, a PF component with the instantaneous physical frequency is obtained. The time and frequency distribution of the original signal can be obtained by the separation of all PF components. For an arbitrary signal x(t), the decomposition process is as follows [18].

Determine all the local extreme points *n_i_* of the original signal *x*(*t*) and calculate the average value *m_i_* of the two extreme points *n_i_* and *n_i_*_+1_. That is:

(1) $$m_{i}=\frac{n_{i}+n_{i+1}}{2}$$

Use local extreme point *n_i_* to calculate envelope estimate *a_i_*:

(2) $$a_{i}=\frac{\left|n_{i}-n_{i+1}\right|}{2}$$

Separate the local mean function *m*_11_(*t*) from the original signal *x(t)*. That is:

(3) $$h_{11}(t)=x(t)-m_{11}(t)$$

*h*_11_(*t*) is divided by the envelope estimation function *a*_11_(*t*) to obtain *s*_11_(*t*):

(4) $$s_{11}(t)=h_{11}(t)/a_{11}(t)$$

The conditions for the termination of the iteration are as follows:

(5) $$\underset{n\to \infty }{\lim}a_{1n}(t)=1$$

The envelope signal can be obtained by taking advantage of all envelope estimation functions generated in the following iteration process:

(6) $$a_{1}(t)=a_{11}(t)a_{12}(t)\cdots a_{1n}(t)=\prod_{q=1}^{n}a_{1q}(t)$$

By multiplying the envelope signal *a*_1_(*t*) and the pure FM signal *s*_1n_(*t*), the first PF component of the original signal can be obtained as follows:

(7) $$PF_{1}(t)=a_{1}(t)s_{1n}(t)$$

The first PF component contains the highest frequency component in the original signal. Its instantaneous amplitude is the envelope signal *a*_1_(*t*), and its instantaneous frequency *f*_1_(*t*) can be calculated by the pure FM signal *s*_1n_(*t*). That is:

(8) $$f_{1}(t)=\frac{1}{2\pi }\frac{d[arc\cos(s_{1n}(t))]}{dt}$$

The first PF component PF_1_(*t*) is separated from the original signal *x(t)*, and a new signal *u*_1_(*t*) is obtained. Repeat the above steps with *u*_1_(*t*) as raw data and cycle k times until *u_k_* is a monotonic function:

(9) $$\{\begin{array}{c} u_{1}(t)=x(t)-PF_{1}(t)\\ u_{2}(t)=u_{1}(t)-PF_{2}(t)\\ \cdots \\ u_{k}(t)=u_{k-1}(t)-PF_{k}(t) \end{array}$$

At this point, the original *x*(*t*) is decomposed into the sum of *k* PF components and a monotone function *u_k_*. That is:

(10) $$x(t)=\sum_{p=1}^{k}PF_{p}(t)+u_{k}(t)$$

**Figure 1:**
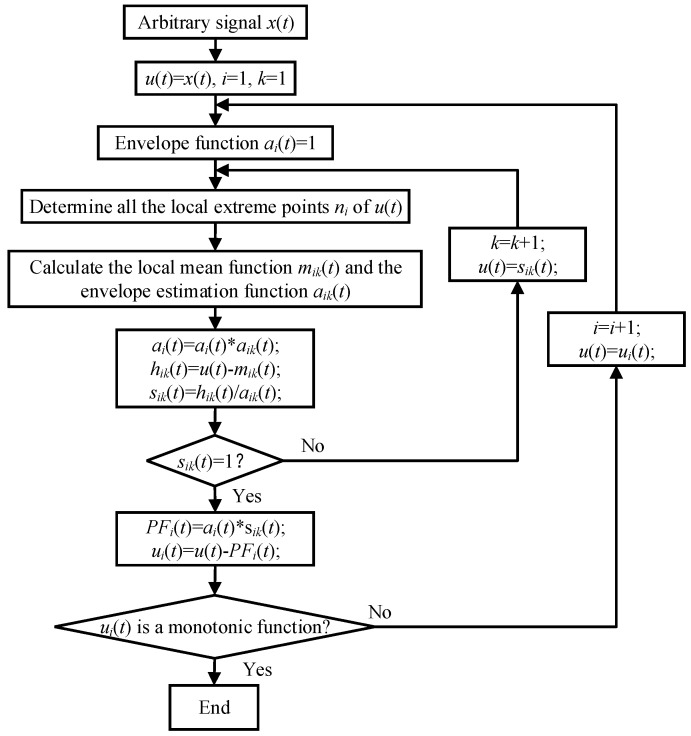
The flowchart of LMD algorithm.

By combining the instantaneous amplitude and instantaneous frequency of all PF components, the complete time-frequency distribution of the original signal *x*(*t*) can be obtained. The flow chart of the whole LMD algorithm is shown in Figure 1.

### 2.2. Stack Denoising Autoencoder

**Figure 2:**
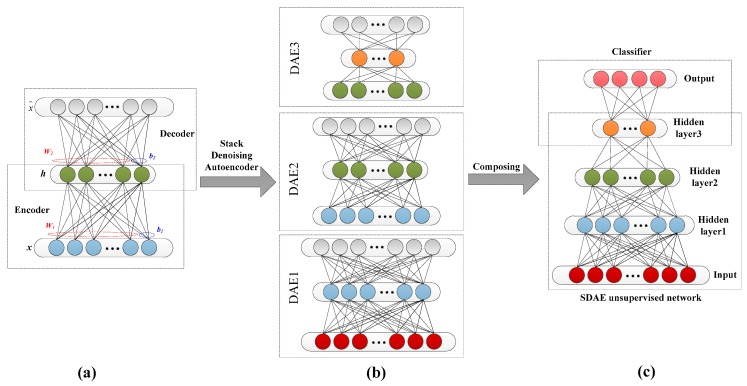
(**a**) Structure of DAE; (**b**) structure of the SDAE; (**c**) supervised for classification.

On the basis of the Autoencoder (AE), the Denoising Autoencoder (DAE) adds noise to the input data in a certain probability distribution, which allows AE learning to remove the noise and reconstruct the signal as much as possible to obtain the input without being disturbed. Therefore, the features learned from noisy inputs are more robust, which improves the generalization ability of the AE model to input data [38]. The DAE structure is shown in Figure 2a.

The Denoising Autoencoder contains two processes.

The encoding process of raw data *X* from the input layer to the hidden layer:

(11) $$h=g\theta_{1}(x)=\sigma (W_{1}x+b_{1})$$

The decoding process from the hidden layer to the output layer:

(12) $$\hat{x}=g\theta_{2}(h)=\sigma (W_{2}h+b_{2})$$

Among them, the mapping parameters are as follows:

(13) $$\theta_{1},\theta_{2}=\arg\min\frac{1}{m}{\sum_{i=1}^{m}\left\|x^{\left(i\right)}-{\hat{x}}^{\left(i\right)}\right\|}^{2}$$

The cost function of DAE is defined as follows:

(14) $$J_{DAE}(W,b)=\frac{1}{m}\sum_{i=1}^{m}\left(\frac{1}{2}{\left\|x^{\left(i\right)}-{\hat{x}}^{\left(i\right)}\right\|}^{2}\right)$$

In order to get the parameters (*W*, *b*) of the DAE neural network, pre-training is carried out. The first hidden layer of the network is trained by using a set of training samples without class labels, and its parameters (*W*_1_, *b*_1_) are obtained. At this point, the first hidden layer of the network converts the original input signal into a vector consisting of hidden unit activation values. Then, the vector is used as the input of the second hidden layer and the second layer parameters (*W*_2_, *b*_2_) are obtained by continued training. Repetitive execution is used to train the output of the front layer as the next input. When training one parameter, the other parameters remain unchanged. After the pre-training process is completed, the parameters of all layers are adjusted simultaneously through the back-propagation algorithm to improve the results. This process is called "fine tuning" to further adjust the features extracted from hidden units [39].

The deep neural network model, stacked by multiple DAEs, is called the Stack Denoising Autoencoder (SDAE). The training of deep network will lead to the disappearance of gradient. Therefore, the principle of greed layer by layer should be adopted. Each layer of the DAE should be trained individually, and the reconstruction error can be minimized. Assuming that each layer of the DAE coding can achieve a better reconstruction effect, the SDAE can achieve high-dimensional feature extraction and dimensionality reduction [40]. The input and output of each layer of the DAE is known to satisfy the normalization requirement, and so a hidden layer encoding vector of the DAE can be used as another DAE input for further coding and dimension reduction, as shown in Figure 2b.

The SDAE is an unsupervised network. Therefore, in order to apply its powerful data processing capability to sample classification, a supervised network is added to the last layer of the SDAE network, and the Soft-max classifier is used to classify the feature vectors, as shown in Figure 2c. The gradient descent method is used to find the optimal parameters in training so that the cost function of Soft-max is minimal to complete the network training [41]. 

## 3. The Proposed Method

The traditional methods of vibration signal denoising usually use filters to set different transmission bands to achieve the suppression or elimination of a particular wave band signal [8,10]. The traditional feature extraction method extracts feature vectors from the original signal by analyzing the time-domain information or frequency domain information and then identifies the state of the machine [11,13]. However, the reciprocating compressor has a complex structure, many internal excitation sources and various forms of movement. Consequently, the vibration signal response presents strong non-stationary and nonlinear characteristics. The traditional method based on classical signal processing technology has some limitations in the fault diagnosis of reciprocating compressors, such as the system signal and vibration signal being difficult to separate, the subjectivity of the artificial feature extraction is stronger, the dimension of the traditional machine learning algorithm is higher, and it faces the problem of the dimensional disaster.

In order to solve the above limitations, a method combining LMD and SDAE is proposed for the intelligent noise reduction and fault diagnosis of reciprocating compressors. The purpose of this method is to de-noise and conduct feature extraction for the signals of a reciprocating compressor valve adaptively and obtain higher classification accuracy. The detailed steps of this method are summarized as follows.

Step 1: Data Acquisition and Noise Reduction Processing. Collect the vibration signal of the reciprocating compressor valve. The collected reference signal is used as the label data of the model, and the training samples of the model are selected. The Gauss white noise signals with different SNRs are added to the collected reference signal, and the vibration signals with different noises are obtained. The single channel vibration signal is decomposed by LMD to get the multiple components. Then, the cross-correlation between the PF components and the reference signal is calculated based on the cross-correlation criterion, and the signal is reconstructed. The virtual noise channel is constructed to reduce the noise of the vibration signal, and the test samples of the model are selected in the reconstructed signal without labels. The Fast Fourier transform (FFT) is applied to the selected training samples and test samples, and the frequency domain signals of the training samples and test samples are obtained. Each sample contains *N* data points. After FFT, each sample will be converted to half of the number of original data points, which is the number of *N*/2+1 points. This will be used as the input of the model in Step 2 and will be fed into the neural network.

Step 2: Model Training. Store the data obtained in step 1, and then train the SDAE neural network model. In order to train the model, the neural network model and its parameters are first initialized. Then, the super parameters in the network model are optimized through loop optimization. Finally, training the neural network through the SDAE using the training samples selected in Step 1 after determining the super parameters of the neural network. If the result of network training does not meet the requirement of the correct rate, the network super parameters will be optimized again. Otherwise, if the result of network training meets the requirement of the correct rate, finish the training and complete the neural network model.

Step 3: Fault Diagnosis. Input the test samples selected without labels in Step 1 into the trained SDAE model in Step 2. And the fault classification and fault diagnosis results of the reciprocating compressor equipment are obtained. The noise data obtained by adding different SNRs to Gauss white noise are processed and diagnosed according to the above steps. Finally, make a comparison of the effectiveness and robustness between the proposed method and the traditional methods.

**Figure 3:**
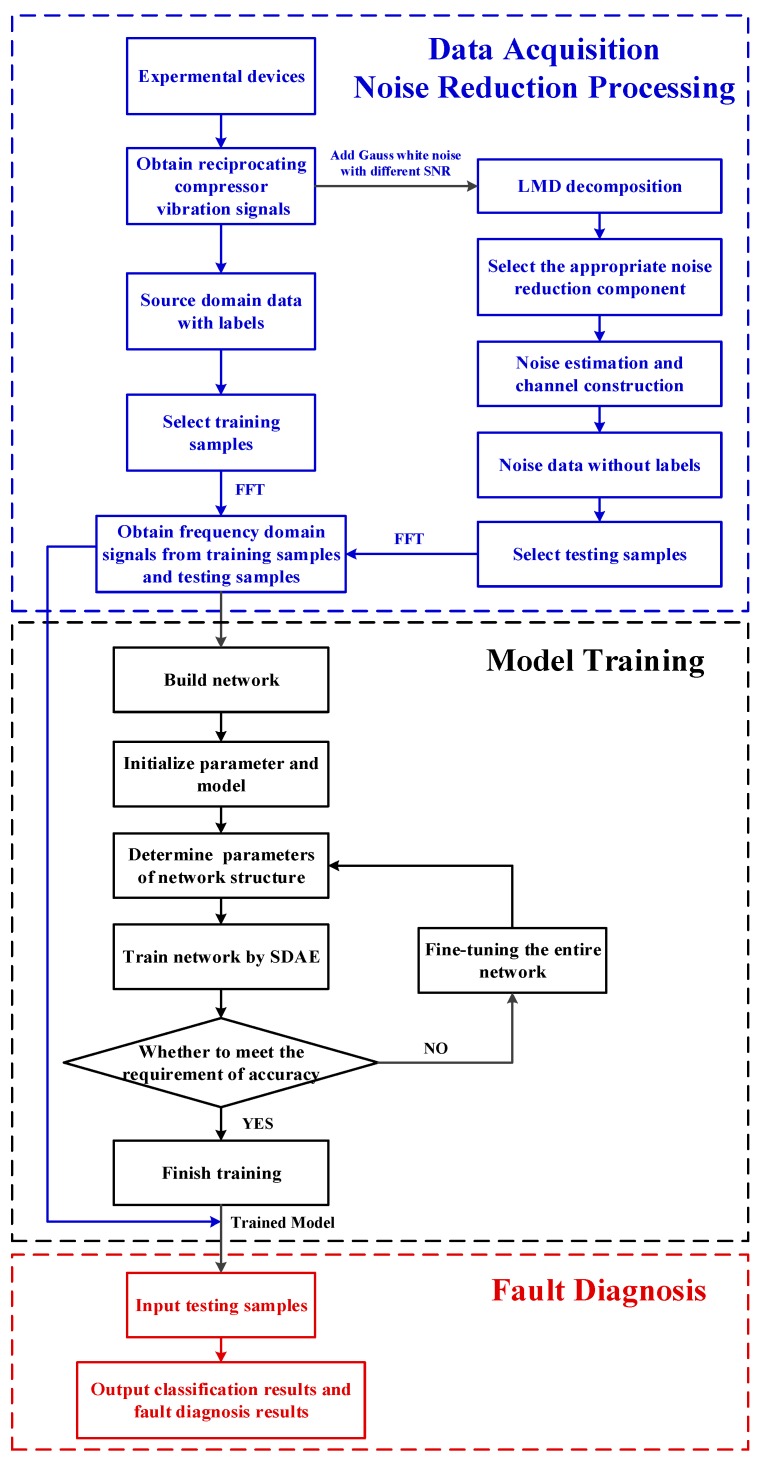
Flowchart of the proposed method.

The flowchart of the proposed reciprocating compressor fault diagnosis method is shown in Figure 3.

## 4. Experimental Studies

### 4.1. Data Description

**Figure 4:**
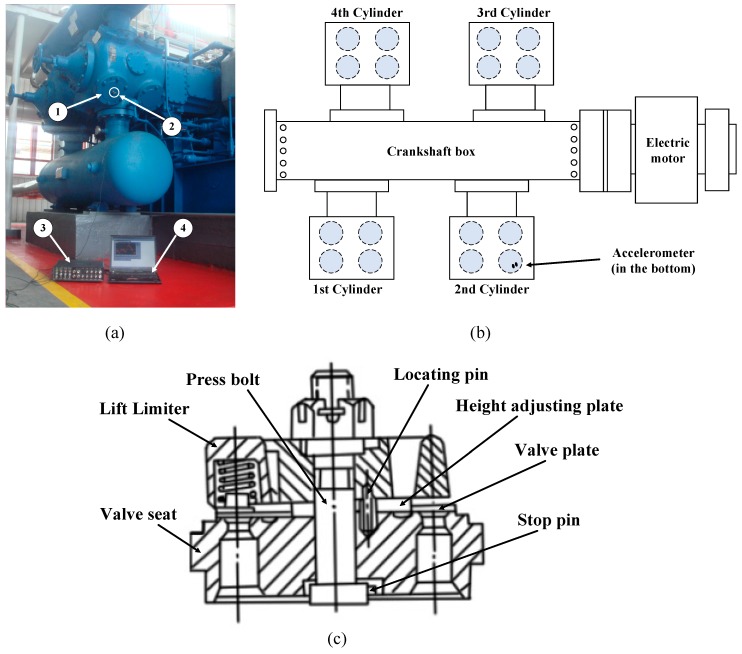
Experimental setup: (**a**) Reciprocating compressor and data acquisition system: 1. exhaust valve, 2. accelerometer on the exhaust valve lid, 3. data acquisition board, 4. control panel. (**b**) Structural sketch of the reciprocating compressor. (**c**) Structural sketch of the exhaust valve.

The research object of this paper is derived from a natural gas reciprocating compressor model WH64 in a petrochemical plant in northwest China. The gas flow of the compressor is 116*104 Nm3/d, and the working pressure is 6.18~6.39 MPa. This compressor has four cylinders and is driven by an electric motor with a rated power of 1,305 kW, as shown in Figure 4a,b. The crankshaft operates at a rotating speed of 993 rpm, that is, the plungers are driven to strike 993 times per minute back and forth. The volume of the cylinder changes with the motion of the plungers, which is the operation principle of this equipment. The intake valve is opened by the increased volume of the cylinder when the plunger moves back; simultaneously, the exhaust valve is closed. Inversely, the exhaust valve is opened, and the intake valve is closed when the plunger moves forth. The mechanism of exhaust valve is as shown in Figure 4c. The valve is susceptible to failure for the frequent movement of the components. The MDES-5 data acquisition system, designed by the China University of Petroleum-Beijing, consists of an accelerometer, a 16-bit data acquisition device, and a computer installed with collecting software. The accelerometer, a piezoelectric acceleration sensor, with the sensitivity of 110 pC/g, is placed on the lid of the exhaust valve in the 2nd cylinder, as shown in Figure 4a. The sampling frequency is set as 16 kHz in this study. 

### 4.2. Fault Diagnosis Using the Proposed Method

**Figure 5:**
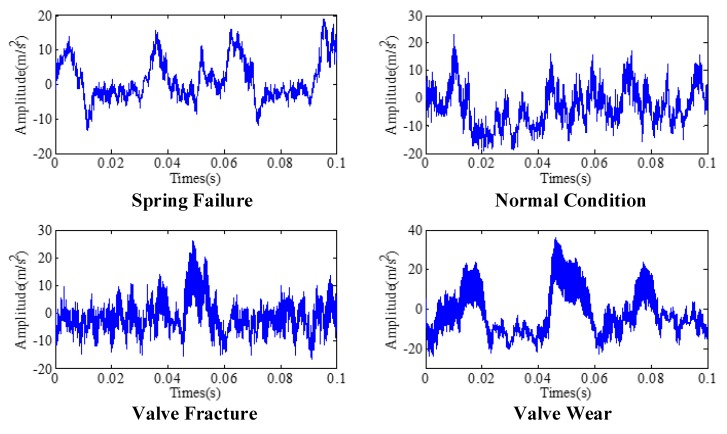
The time-domain waveforms of four datasets

In this experiment, four conditions of reciprocating compressor valves are selected, including Spring Failure (SF), Normal Condition (NL), Valve Fracture (VF) and Valve Wear (VW). The time-domain waveforms of them are depicted in Figure 5. 

The number of samples selected for the training datasets and testing datasets and the length of samples are shown in Table 1.

The signal-to-noise ratio (SNR) is an important parameter that describes the proportional relationship between the active component and the noise component in the signal. The unit of SNR is dB. The SNR is defined as follows:

(15) $$\mathrm{SNR}=\frac{\mathrm{Signal}\;\mathrm{energy}}{\mathrm{Noise}\;\mathrm{energy}}={\left(\frac{\mathrm{Pure}\;\mathrm{signal}}{\mathrm{Noise}\;\mathrm{signal}\;-\;\mathrm{Pure}\;\mathrm{signal}}\right)}^{2}$$

**Figure 6:**
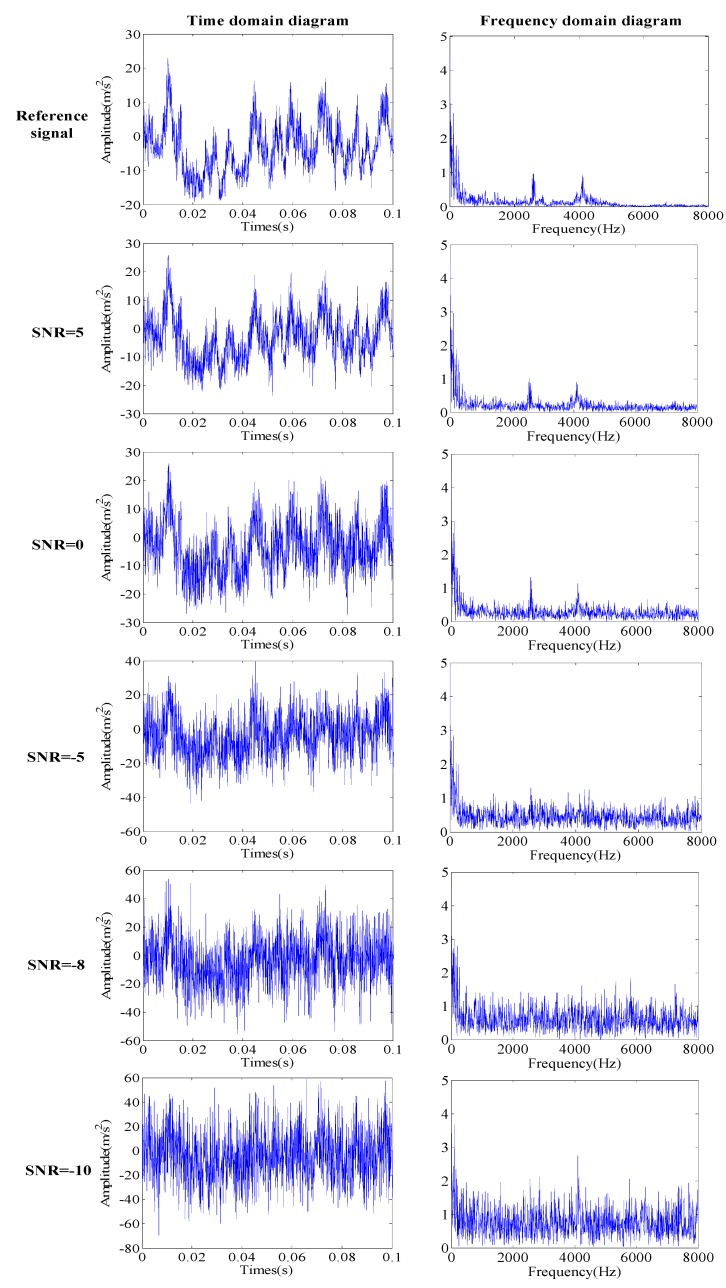
Time domain and frequency domain diagrams of different noise signals.

In order to prove the noise reduction ability of the proposed method, the signal collected in the field is taken as the reference signal, and Gaussian white noise with different SNRs is added to the reference signal. First, the reference signals of the four conditions of the valve faults of the reciprocating compressor are obtained. On adding the Gauss white noise with SNRs of 5, 0, -5, -8 and -10 to the reference signal, different noise signal types are obtained. Taking the normal sample data of the valve as an example, the time domain and frequency domain diagram of the reference signal and noise signal with different SNRs are drawn, as shown in Figure 6.

**Figure 7:**
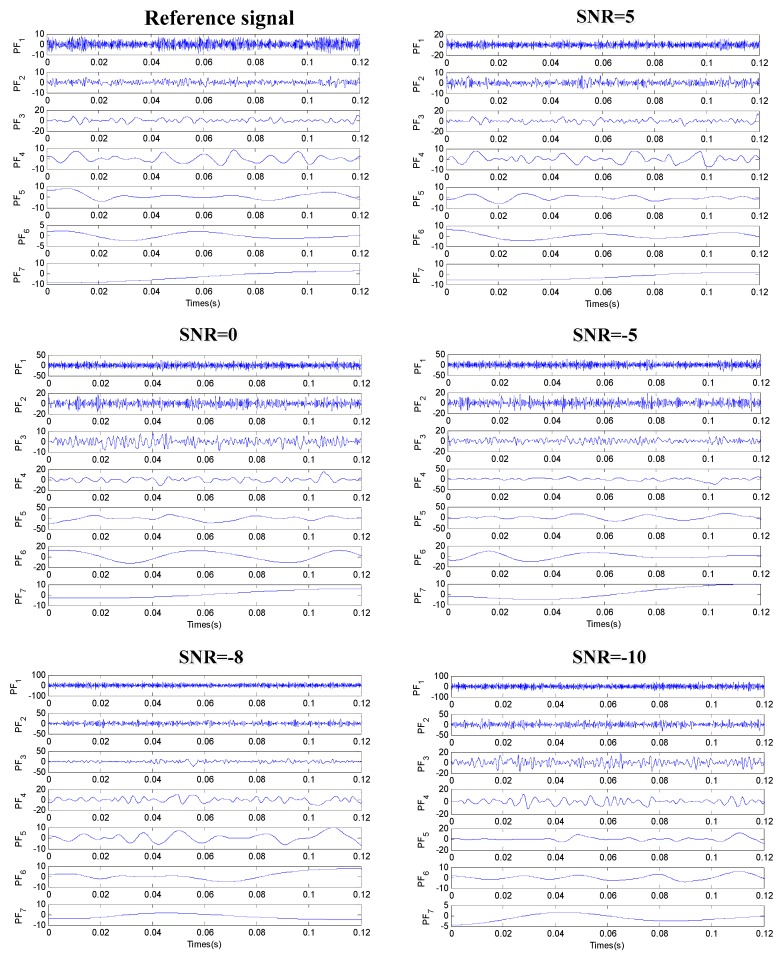
PF components after the decomposition of different noise signals.

**Figure 8:**
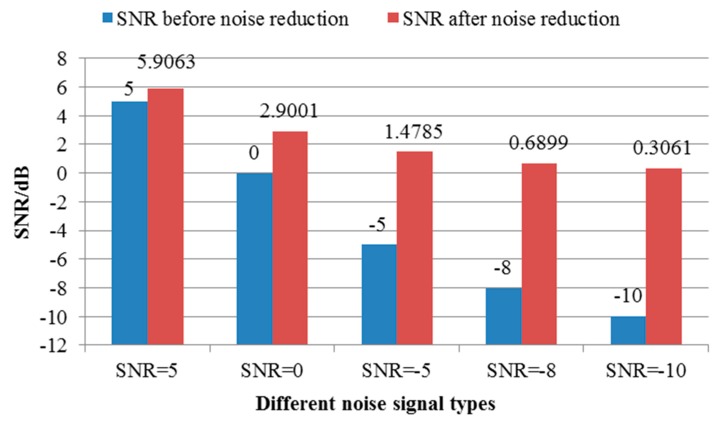
SNR collation map before and after noise reduction.

After the noise signal of different SNRs is obtained, the signal processing method of LMD is used to decompose the single channel vibration signal and get multiple PF components, as shown in Figure 7. Then, the cross-correlation between each PF component and the reference signal is calculated based on the cross-correlation criterion. The cross-correlation coefficient of PF components and the reference signal is shown in Table 2. The vibration signal is reconstructed according to the calculated cross-correlation coefficient, and the virtual noise channel is constructed to reduce the noise of the vibration signal. The SNRs before and after noise reduction are shown in Figure 8.

The data of the four conditions of valves of the reciprocating compressor are processed by noise reduction and stored. The 2400 training samples and 800 testing samples are selected from each condition. The SDAE neural network model is established, the training samples are input into the SDAE model, and the sample data are trained and iterated through Matlab 2013a. The neural network model with the highest accuracy of sample classification is found in all training results. Then, the optimal neural network super parameters of the SDAE network are determined. Finally, the neural network super parameters determined by parameter optimization are shown in Table 3.

**Figure 9:**
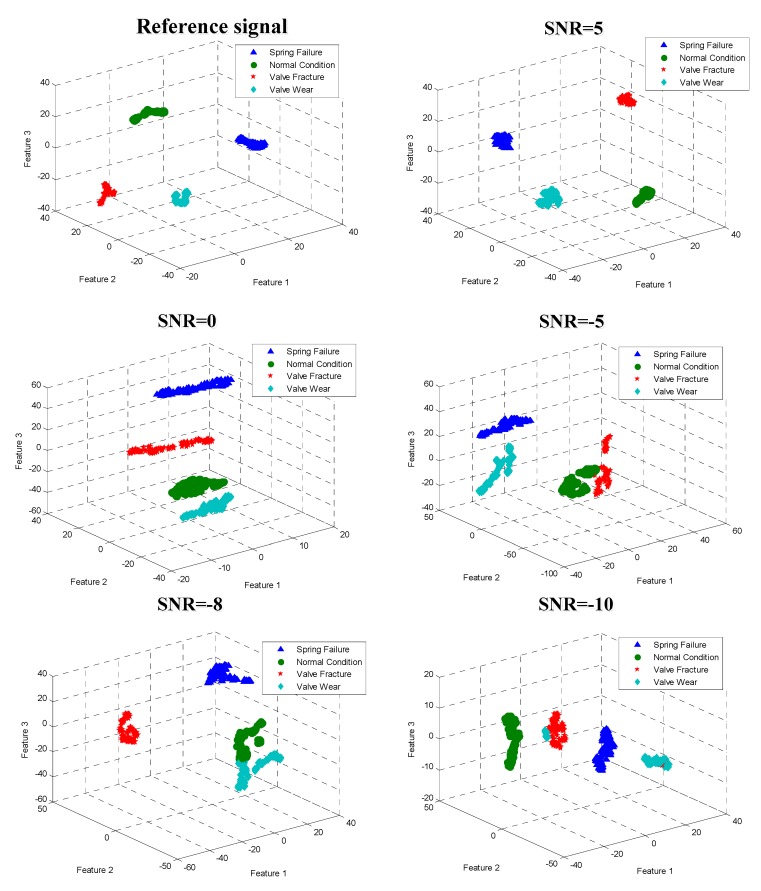
Feature extraction of different noise signals.

The optimal neural network structure chosen after training is 1024-500-250-50. That is, the neural network has four layers, including one input layer, two middle hidden layers, and one output hidden layer, to predict the classification. According to Section 2.2, the SDAE network is a stack of multiple DAE networks, and so the structure of this neural network is made up of three DAEs, which are 1024-500-1024, 500-250-500 and 250-50-250. The unlabeled testing data is input into the trained model, and the features of the testing samples are extracted, as shown in Figure 9.

The features of the vibration signal, which contain six different SNRs, are extracted, and these features are reduced from 50 dimensions to 3 dimensions. From Figure 9, it can be seen that the effect of the feature extraction for the reference signal is the best. The four conditions of faults are far from each other, and there is no overlap. With the decrease in SNR, the extracted features are gradually overlapped. However, the four conditions of faults can still be distinguished from each other, and the overall effect is ideal.

To evaluate the performance of the proposed method, the classification accuracy is defined as follows:

(16) $$\mathrm{acc}uracy=\frac{N^{\prime }}{N}$$

where *N’* is the number of correctly classified samples and *N* is the total number of samples.

**Figure 10:**
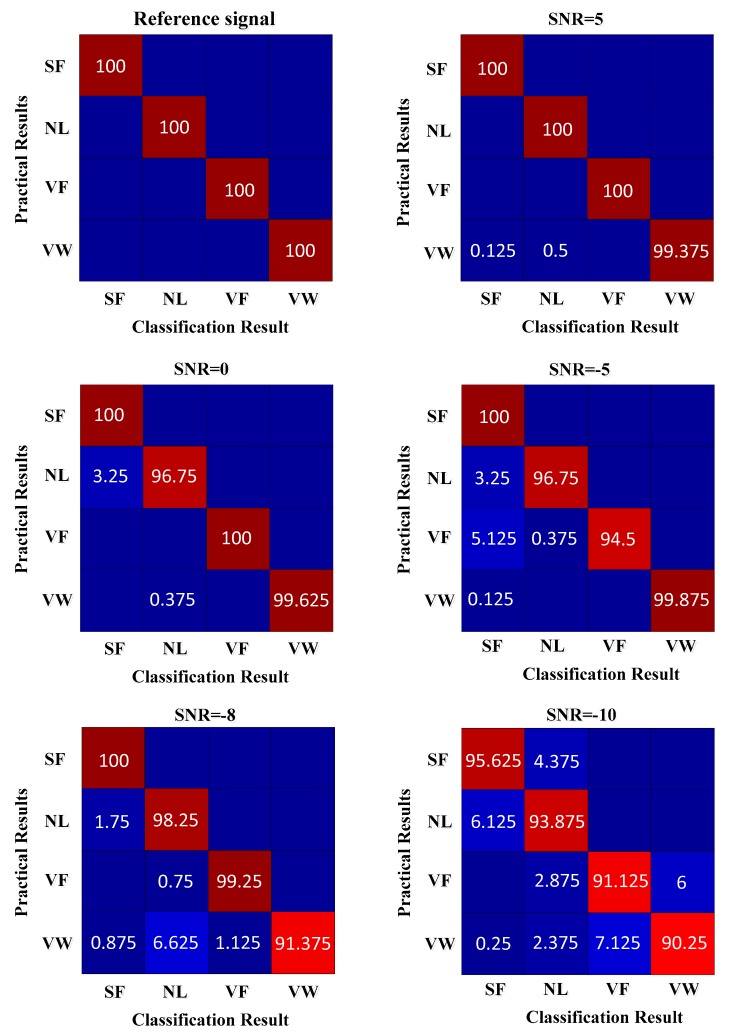
Classification results.

Figure 10 illustrates the classification results. The abscissa is the classification result, and the ordinate is the practical result. The classification results in the diagonal line are the classification accuracy, and the remainder is the misclassification rate.

From Figure 10, the accuracy rate of the four conditions of fault classification is 100% when there is no noise. When the SNR is 5 dB, 0.125% of the samples of valve wear are misclassified as spring failure, 0.5% of the samples are misclassified into the normal condition, and 99.375% of the samples are correctly classified. When the SNR is −5 dB, the samples of spring failure are all correctly classified. A total of 3.25% of the samples of the normal condition are misclassified as spring failure, 5.125% of the samples of valve fracture are misclassified as spring failure, and 0.375% of the samples are misclassified into the normal condition. In addition, 0.125% of the samples of valve wear are misclassified as spring failure. When the SNR is −10 dB, 95.625% of the samples of spring failure are correctly classified, 93.875% of the samples of the normal condition are correctly classified, 91.125% of the samples of value fracture are correctly classified, and 90.25% of the samples of value wear are correctly classified. It can be seen that, under the condition of large noise interference, the noise reduction and fault diagnosis results of the proposed method are ideal.

### 4.3. Comparison and Analysis of Traditional Methods

**Figure 11:**
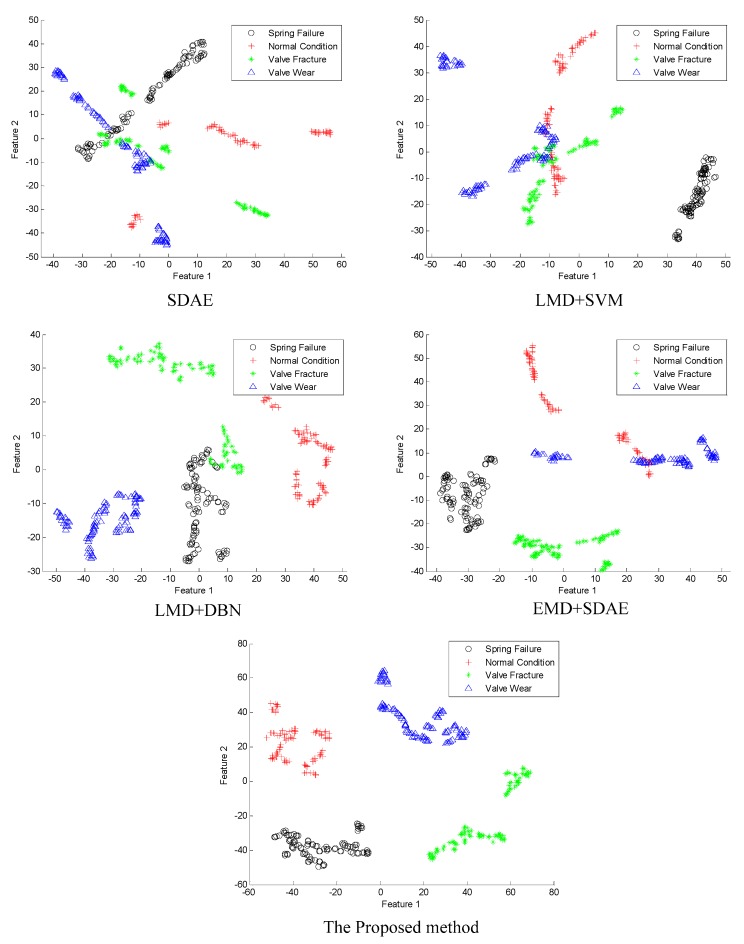
Fault feature extraction chart of the valves in different methods.

In order to verify the effectiveness, the proposed method is compared with the other four methods, including the SDAE, the combination of LMD and SVM, the combination of LMD and DBN, and the combination of EMD and SDAE. All five methods are used to extract the features of the four conditions of valve faults when the SNR is −10 dB; the feature extraction effects are as shown in Figure 11. 

From Figure 11, when the SNR is −10 dB, the effect of feature extraction using SDAE is negative because the four conditions of faults are scattered everywhere and the spring failure, valve fracture and valve wear are partially overlapped and cannot be separated. The method combining LMD and SVM can separate the spring failure, but the normal condition, valve fracture and valve wear are partially overlapped, and the effect of separation is not ideal. The method combining LMD and DBN can separate the normal condition and valve wear, but only the clustering result of the valve wear features is preferable, and the spring failure and valve fracture are partially overlapped. The method combining EMD and SDAE can separate the spring failure and value fracture, but the normal valve and valve wear are scattered and partially overlapped. The proposed method in this paper can separate the four conditions of faults well, and the distances of the features that are extracted are far away from each other. Although the result of clustering does not reach the ideal state, the effect of feature extraction has been significantly improved by comparing it with the other four methods.

In this study, the ratios of the correct sample number to the total sample number are used for the classification accuracy, as described in Equation (16). The classification results of several cases are obtained by the above five methods to prove that the proposed method is robust and effective for different SNR noise signals, and the correct rate average of 3200 testing samples for four fault types is considered as the overall classification accuracy, as shown in Table 4.

**Figure 12:**
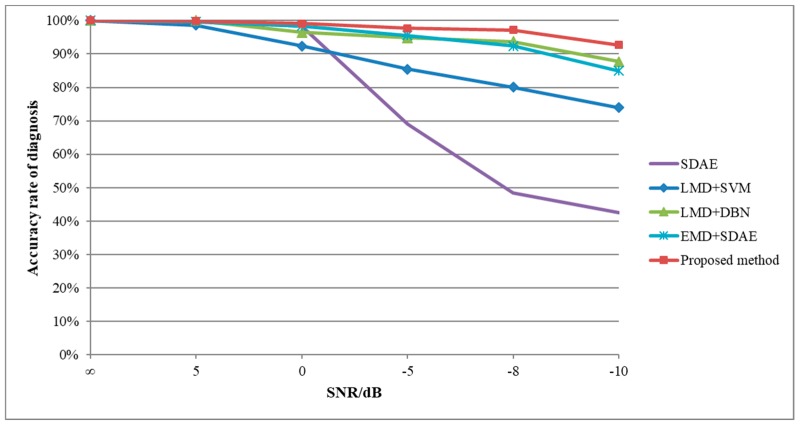
Compressor diagnosis results of the employed methods with different SNRs.

From the diagnosis results, all methods show good results when the SNR is positive infinity. With the continuous reduction of the SNR, there are different downward trends in various methods, as shown in Figure 12.

Taking the average classification results when the SNR is −10 dB as an example, the diagnostic results of SDAE, LMD+SVM, LMD+DBN, EMD+SDAE and the proposed method are 42.56%, 73.97%, 87.69%, 84.97%, and 92.72%, respectively. In the case of an extremely low SNR, the proposed method improves the classification accuracy by 5 percentage points compared with the traditional methods, and the robustness and effectiveness of the proposed method are proved.

## 5. Conclusions

In view of the fact that the SNR of the vibration signal is too low in industrial production, and the traditional machine learning methods have some limitations, such as the system signal and vibration signal are difficult to be separated, the subjectivity of the artificial feature extraction is stronger, and the traditional machine learning algorithm is faced with the problem of the dimensional disaster, this paper proposed an intelligent diagnosis method combining LMD and SDAE to solve the above problems. The main conclusions can be drawn as follows.

(1) The proposed method reduces the overlapping of the system signal and the noise signal by decomposition and reconstruction. Therefore, the proposed method has strong noise reduction effect and the obtained de-noised signal has a high SNR.
(2) The proposed method utilizes the massive data to fully explore the information of vibration signals, and learn the high-dimensional deep features. Therefore, the proposed method has good learning ability and the necessary generalization abilities.
(3) The features of the de-noised signal are automatically extracted by the proposed method, which reduced the subjectivity of artificial features extraction. Therefore, the proposed method has a better feature extraction effect and higher diagnosis accuracy, which proves the effectiveness of the proposed method in adaptive feature learning.
(4) The proposed method has great application prospects in the fault diagnosis of industrial reciprocating compressors based on the experimental results of this study. Especially, this method has good effect of adaptive feature extraction under low SNR, and the accuracy of the classification diagnosis is higher than that of traditional fault diagnosis methods, which proves the robustness of the proposed method.

This paper focuses on the intelligent method of noise reduction and fault diagnosis. A neural network structure with a relatively good training effect is used for data processing and interpretation. In future studies, optimization of the network structure can be further carried out and better network super parameters can be searched to fully explore the potential of the proposed method. Moreover, this paper focuses on the failure patterns of the valve; the failure problems of other reciprocating compressor components (such as piston, rod, bearing and rotor) will be further studied in the future.

## Figures and Tables

**Table 1:** Information of the training datasets and testing datasets.

Datasets	Training Datasets		Testing Datasets		Label
	Number of Samples	Length of Samples	Number of Samples	Length of Samples	
**SF**	2400	2048	800	2048	1
NL	2400	2048	800	2048	2
VF	2400	2048	800	2048	3
VW	2400	2048	800	2048	4

**Table 2:** Cross-correlation coefficient between each component and the reference signal of the normal condition.

Signal Type	PF1	PF2	PF3	PF4	PF5	PF6	PF7
Reference signal	0.4022	0.2807	0.4055	0.4760	0.3716	0.2540	0.3551
SNR = 5	0.2652	0.2007	0.4591	0.4759	0.3117	0.4504	0.3816
SNR = 0	0.1364	0.1195	0.0635	0.3442	0.5348	0.4334	0.2269
SNR = −5	0.1416	0.0047	0.0905	0.2285	0.6616	0.4186	0.2204
SNR = −8	0.0900	0.1516	0.0950	0.3605	0.4939	0.1637	0.0423
SNR = −10	0.0962	0.1345	0.0871	0.1815	0.5480	0.4221	0.2025

**Table 3:** Information of neural network super parameters.

Signal Type	Network Structure	Opts. Numepochs	Opts. Batchsize	Learning Rate	Feature Dimension
Reference signal	1024-500-250-50	50	10	1	50
SNR = 5	1024-500-250-50	40	20	1	50
SNR = 0	1024-500-250-50	80	50	1	50
SNR = −5	1024-500-250-50	100	100	1	50
SNR = −8	1024-500-250-50	240	100	1	50
SNR = −10	1024-500-250-50	350	100	1	50

**Table 4:** Classification accuracy results by different methods.

SNR (dB)	SDAE	LMD+SVM	LMD+DBN	EMD+SDAE	The Proposed Method
∞	100%	100%	100%	100%	100%
5	100%	98.63%	100%	99.66%	99.84%
0	98.28%	92.44%	96.47%	98.44%	99.09%
−5	68.91%	85.47%	94.84%	95.59%	97.75%
−8	48.47%	80.06%	93.72%	92.44%	97.22%
−10	42.56%	73.97%	87.69%	84.97%	92.72%
